# Banana split: biomass splitting with flash light irradiation†

**DOI:** 10.1039/d1sc06322g

**Published:** 2022-01-25

**Authors:** Wanderson O. Silva, Bhawna Nagar, Mathieu Soutrenon, Hubert H. Girault

**Affiliations:** Laboratory of Physical and Analytical Electrochemistry, École Polytechnique Fedérale de Lausanne (EPFL) Valais Wallis CH-1950 Sion Switzerland wanderson.oliveiradasilva@epfl.ch; Institute of Systems Engineering, HES-SO Valais-Wallis CH-1950 Sion Switzerland

## Abstract

Biomass splitting into gases and solids using flash light irradiation is introduced as an efficient photo-thermal process to photo-pyrolyze dried natural biomass powders to valuable syngas and conductive porous carbon (biochar). The photo-thermal reactions are carried out in a few milliseconds (14.5 ms) by using a high-power Xenon flash lamp. Here, dried banana peel is used as a model system and each kg of dried biomass generates *ca.* 100 L of hydrogen and 330 g of biochar. Carbon monoxide and some light hydrocarbons are also generated providing a further increase in the high heating value (HHV) with an energy balance output of 4.09 MJ per kg of dried biomass. Therefore, biomass photo-pyrolysis by flash light irradiation is proposed as a new approach not only to convert natural biomass wastes into energy, such as hydrogen, but also for carbon mitigation, which can be stored or used as biochar.

## Abstract 2

Biomass splitting into gases and solids using flash light irradiation is introduced as an efficient photo-thermal process to photo-pyrolyze dried natural biomass powders to valuable syngas and conductive porous carbon (biochar).

## Graphical abstract

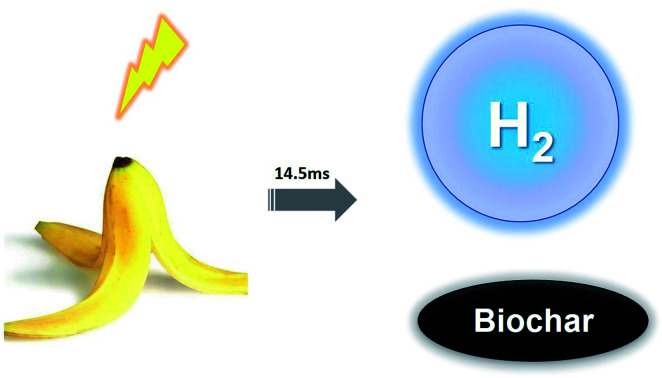

## Introduction

Over the last few decades, the high consumption of energy from fossil fuels has promoted a massive increase of greenhouse gas emissions worldwide (*e.g.* carbon dioxide, methane, *etc.*), leading to several environmental impacts such as global warming and climate change.^1,2^ The use of renewable energies is being pursued globally to alleviate these problems.

Natural biomass, and in particular wood, has historically been a major source of energy. Biomass originating from organic carbon-based plants or animals is also a source of carbon capture and storage. During its lifetime, biomass absorbs CO_2_ and hence removes carbon from the atmosphere to store it, and its decomposition could result in negative emission technologies or greenhouse removal technologies as long as no CO_2_ or CH_4_ is generated.

Biomass decomposition at elevated temperatures, or thermochemical conversion, creates valuable combustible gases and biofuels that can be used for energy applications. Alternatively, the majority of the carbon content can be converted into solid carbon often referred to as biochar that can be stored safely, for example as a valuable amendment in soil.^3^

Gasification and pyrolysis are the main routes currently applied for biomass thermochemical conversion. Biomass gasification is a process that converts solid/liquid organic sources, usually from natural biomass wastes, into gaseous and solid compounds, which normally takes place at approximately 1000 °C. The gas phase, denoted as syngas, is a gas mixture of hydrogen, methane, carbon monoxide, carbon dioxide and some light hydrocarbons,^4^ and is normally used for power generation or biofuel production, and the solid phase denoted as “char” or “biochar” is a mixture of mainly carbon with a small unconverted organic fraction and ash.^5^

Biomass pyrolysis is another route to convert biomass, which is similar to the gasification process; however it is carried out at lower temperatures (between 400 and 800 °C) and pressure up to 5 bar in the absence of an oxidizing agent.^4,6^ It can usually be divided into three categories: (i) conventional pyrolysis, (ii) fast pyrolysis and (iii) flash pyrolysis. Conventional pyrolysis is done at temperatures below 450 °C and normally provides a high content of charcoal. Fast pyrolysis is carried out at 450–600 °C and can yield bio-oil up to 75% with a high heating rate (300 °C min^−1^) and a short residence time (usually 2 s).^7^ Flash pyrolysis is a very fast pyrolysis process that is carried out up to 600 °C and 1000 °C min^−1^ with a residence time lower than 1 s, and it is normally adopted to maximize the gas generation.^7^ However, one of the disadvantages of pyrolysis is the need for specific reactors required to accommodate high temperature and pressure environments.

Biological routes for biogas generation from biomass include anaerobic digestion and fermentation, in which, microorganisms are used to breakdown the bio-feedstock for producing biofuels (bioethanol or butanol) or combustible gases such as CH_4_, CO_2_, H_2_*etc.* After the conversion, the digested waste is used for additional carbon acquisition processes or simply incinerated. Although this approach is used at the industrial level, it can cause some problems for waste water management.^8,9^ Those routes are slower than thermochemical conversion.

Flash light photonic surface treatment or photonic curing is a process that produces full spectrum white light from an electric arc lamp and is typically used for thermal annealing. Recently, it has been used in electronics for sintering metallic inks into conductive tracks over thermally sensitive substrates. In this technique, powerful flashes can deliver high broad wavelength light pulses in a short exposure time (<1 ms), which promotes not only the heating and evaporation of residues, wet solvents, polymers and binders, but can also be used to carry out reactions such as the reduction of metal ions or oxides to bulk metal, and then generate conductive films or tracks for example of silver^10,11^ or copper.^12–14^ In this process, the temperature of the metal particles absorbing light increases hundreds of degrees, but only locally and for a very short time, and hence a wide range of transparent low glass transition temperature polymer materials such as PET or PEN can be used as substrates with no damage.

Our laboratory has recently developed different reactive surface processes based on photo-thermal non-equilibrium reactions using the same white flash light from a xenon flash lamp. For example, it has been used to modify the surfaces of materials such as graphene oxide to form conductive graphene^15^ and also metal oxides to generate metal carbides.^16^ Flash light photonic surface reactions have also been recently introduced to generate metallic nanoparticles from metal salt precursors, for example Pt, Ni, NiFe, Au, Ag and Au–Ag or even metal complexes such as Prussian blue.^17–19^

In order to take advantage of the high-power energy source provided by a xenon flash lamp and also short pulses to promote photo-thermal reactions, we introduce here a new and rapid protocol that converts dried natural biomass powders into syngas and biochar. The main principle of this approach is to generate a powerful flash light shot (from a photonic curing system), which is absorbed by biomass, instantaneously promoting photo-thermal biomass conversion into syngas and biochar. Here, the process is carried out in a stainless-steel reactor with a standard glass window at near ambient pressure and under an inert atmosphere (argon). This approach not only can minimize the time consumed compared to conventional pyrolysis processes but can also maximize the syngas yield, in particular H_2_. The other added advantage is the amount of solid carbon biochar produced, which reaches 33 wt% of the original dried banana peel mass.

## Experimental

**Fig. 1:**
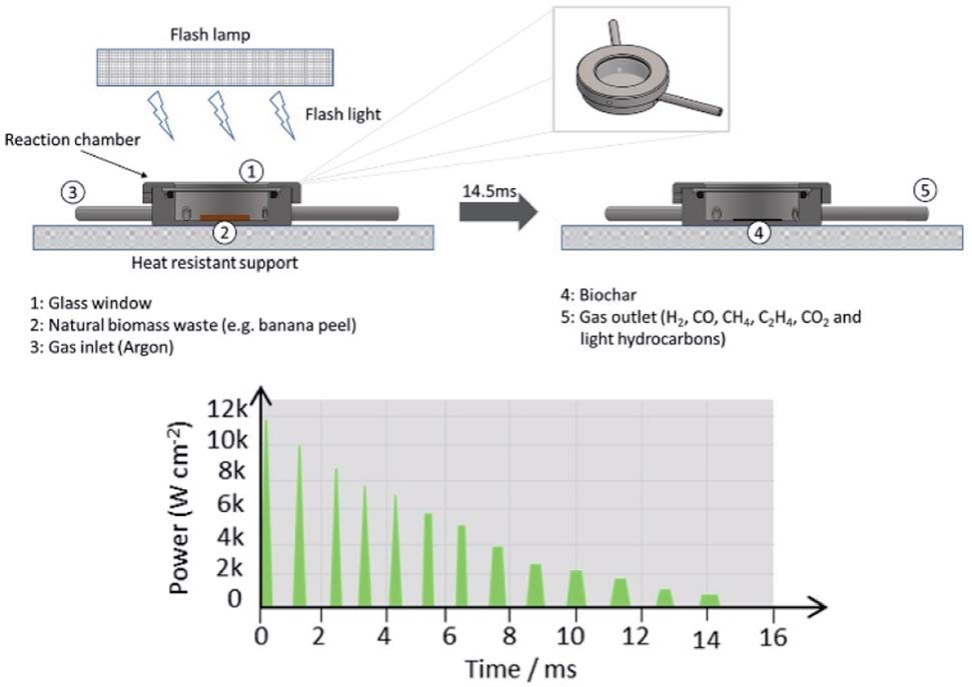
Schematic representation of the flash light irradiation system designed for the pyrolysis of natural biomass wastes, and flash power profile of a 575 V flash shot constituted of 13 microsecond flashes for a total duration of 14.5 ms.

Fig. 1 shows a generic scheme of the flash light irradiation system employed to photo-pyrolyze natural biomass wastes. Here, a simple reaction chamber manufactured from stainless steel is designed with one inlet and outlet (insets 3 and 5) to control the gas atmosphere and perform the flash photo-pyrolysis process without oxygen. Firstly, a thin layer of biomass (2 and 10 mg with a particle size of 20 μm) is deposited onto a substrate (stainless steel, glass and/or glassy carbon) and secondly placed in a reaction chamber and sealed with a standard glass window (inset 1) of 1 mm thickness. Thirdly, the full apparatus is placed over a heat resistant support/table. The air from the chamber is replaced by an inert atmosphere (argon) and exposed directly to flash light irradiation by using a xenon lamp, from a PulseForge 1300 photonic curing system (Novacentrix, USA) with a xenon flashlamp as illustrated in Fig. S1†, selecting a desired charging voltage, exposure time, pulse shape, number of micropulses, repetition rate, *etc.* The emission spectrum of the Xenon lamp is also presented. Here, each apparent single flash shot is composed of 13 repeated microsecond flashes as illustrated in Fig. 1, which corresponds for example in the case of a 575 V-pulse to a total energy density of 13.1 J cm^−2^ for 14.5 ms. Most of the tests were performed with 575 V-pulses with 5 consecutive flash shots providing a total reaction time of 12 s, as each flash shot is separated by a period of 3 s to recharge the capacitors. Syngas and biochar are generated as by-products (insets 4 and 5).

Due to the fast process, simulated temperature values for the deposited biomass films were estimated by using SimPulse software from Novacentrix with inputs such as bulk material properties, the thickness of the biomass layer and substrate properties.^20^

Banana peel, corncob, orange peel, coffee bean and coconut shell were selected as biomass sources, and they were initially dried at 105 °C for 24 h for water and moisture removal and then ground and sieved to obtain a thin powder. Gas analyses were initially carried out by using a PrismaPlus mass spectrometer (OMNISTAR GSD 320, Pfeifer) with an yttriated iridium filament to identify qualitatively the gaseous products generated from the flash light photo-pyrolysis of the five different natural biomass wastes. Here, a metallic tube outlet containing all generated gases, Fig. 1 and inset 5, was directly connected to the MS gas inlet equipped with a stainless-steel capillary (OmniStar, stainless steel, 1 m temperature-regulated gas sampling line, and 200 °C) and then analyzed in multiple ion detection mode. MS tests were performed under a controlled argon flow and all gaseous products were monitored at different values of *m*/*z* (mass/charge) ionic signals. Then, gas chromatography analyses were performed to quantify the gaseous products by using a Micro-GC 490 (Agilent) with a molecular sieve 5A and PoraPLOT U columns and argon and helium gases as mobile phases. Additional Micro-GC instrument conditions are described in Table S1 (ESI).† Scanning electron microscopy (SEM) images were collected from a FEI Tecnai Osiris instrument. X-ray photoelectron spectroscopy (XPS) analyses were carried out by using a Physical Electronics VersaProbe II X-ray photoelectron spectroscope. CasaXPS software was used to fit all XPS spectra. Raman spectra were collected by using an inVia confocal Raman microscope (Renishaw) with a green laser.

## Results and discussion

A total dry matter of 10.2 wt% was obtained from fresh banana peel after 24 h at 105 °C. This dried powder photo-pyrolyzed from flash light irradiation with 575 V-pulses and 5 consecutive flash shots over 12 s generated a final carbonized matter of 33 wt%, here biochar. The main weight (57 wt%) corresponds to gaseous compounds generated at the high temperature reached in a few milliseconds from the high intensity flash light shot. The remaining 10 wt% is supposed to be lost, for instance materials deposited on the chamber surface and in the connecting pipes. The elemental analysis of the banana peel powder presented in Table S2 (ESI)† evidenced carbon and oxygen as the major elements corresponding to *ca.* 93.7 wt%, which are attributed mainly to biomass components (lignin, cellulose, hemicellulose, *etc.*), and the remain percentage of 6.3 wt% corresponds to hydrogen, nitrogen and sulfur as described previously.^21,22^

**Fig. 2:**
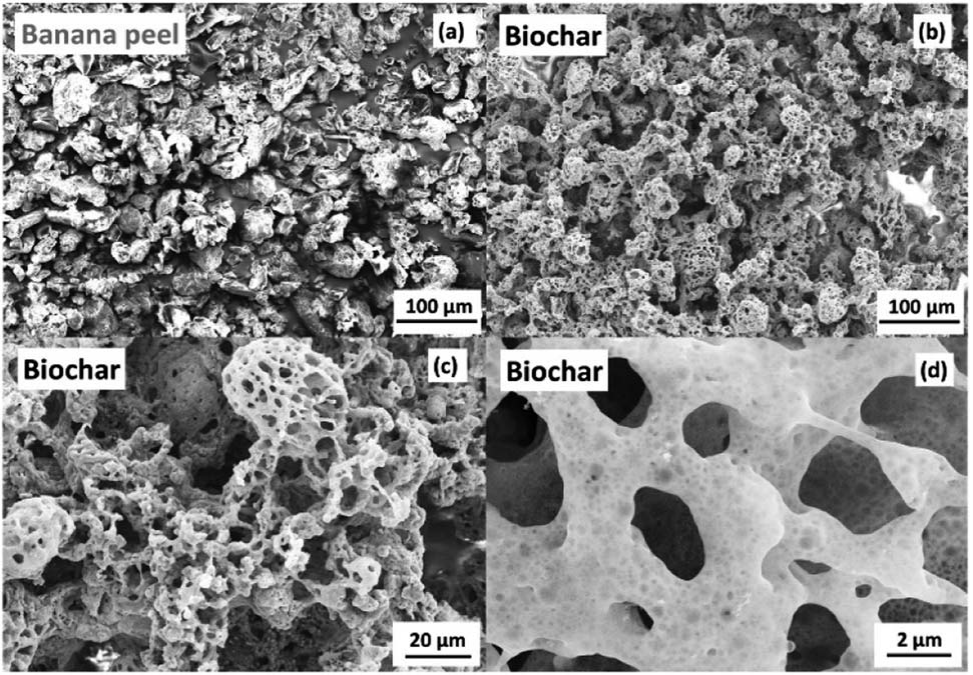
Scanning electron microscopy images of (a) banana peel powder and the respective (b), (c) and (d) biochar generated from flash light photo-pyrolysis at 575 V-pulses and 5 flash shots.

Scanning electron microscopy images presented in Fig. 2 show highly porous structures (Fig. 2b–d) compared to the banana peel powder (Fig. 2a), which can be attributed to the fast photo-heating process and gas release promoted by the high flash light intensity. Fig. S2 (ESI)† presents Raman and XPS spectra for the porous carbon generated from the photo-pyrolysis of the banana peel. The Raman spectrum in Fig. S2a (ESI)† shows characteristic peaks at 1345 and 1590 cm^−1^ that correspond to the D and G-bands of carbon, respectively, and the D-band is attributed to the disordered graphitic structure, while the G-band (sp^2^ hybridized carbon) is ascribed to an ideal graphitic structure. The *I*_D_/*I*_G_ ratio of 0.97 calculated from D and G-band intensities suggests a high degree of graphitic defects. The XPS analysis of the biochar in Fig. S2b (ESI)† shows a typical C 1s XPS spectrum deconvoluted into three contributions centered at 284.9, 286.7 and 289.1 eV corresponding to the C–C/C=C (sp^3^ and sp^2^ carbon), C–O and C=O groups, respectively. Fig. S2c and d (ESI)† present K 2p and O 1s XPS spectra fitted into components attributed to potassium carbonate and/or oxides and O=C, O–C and O=C–OH groups, respectively.^23^

**Fig. 3:**
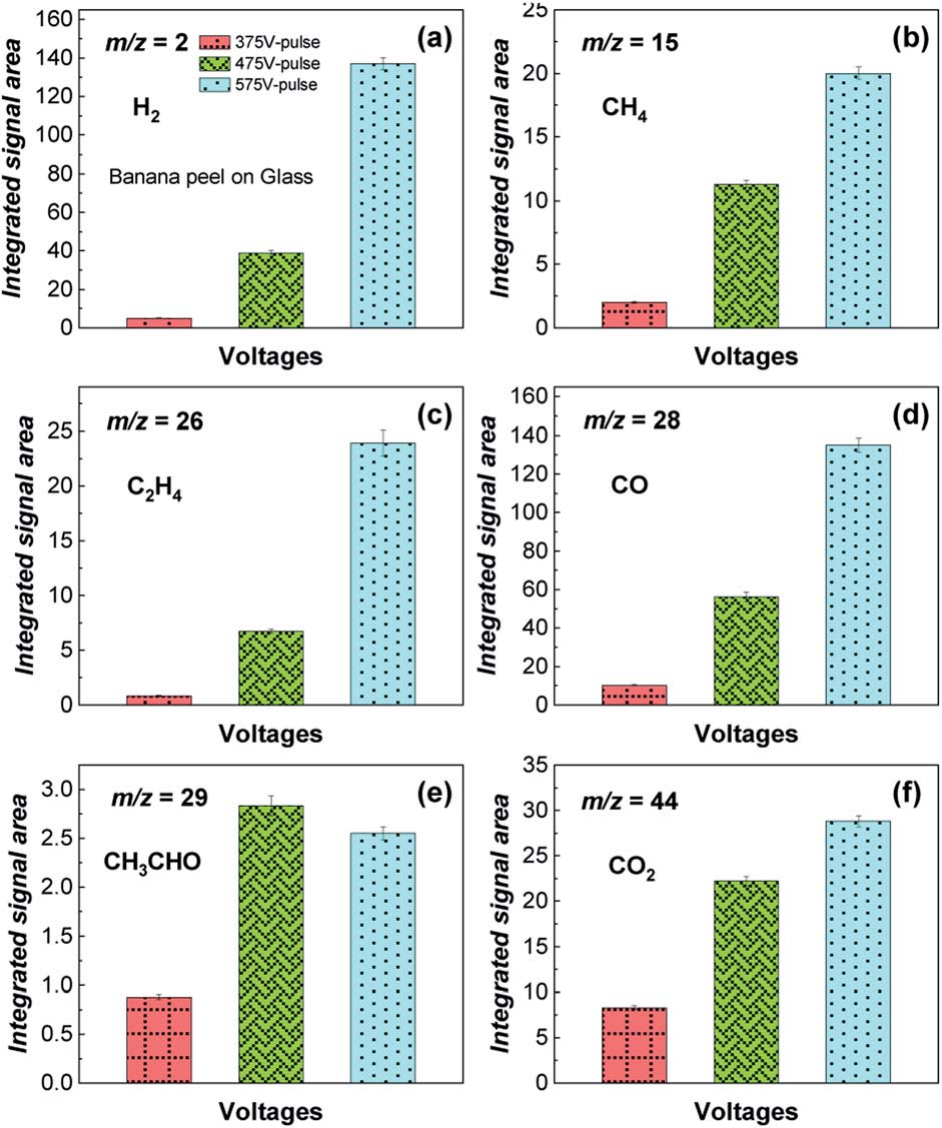
(a–f) Integrated ionic currents for *m*/*z* 2 (H_2_), 15 (CH_4_), 26 (C_2_H_4_), 28 (CO), 29 (CH_3_CHO) and 44 (CO_2_) obtained during the photo-pyrolysis of banana peel on a glass substrate by flash light irradiation for 12 s with 5 flash shots at different voltages: 375 V-pulse, 475 V-pulse and 575 V-pulse.

Fig. 3 shows qualitative experimental analysis performed by mass spectrometry (MS) of the gaseous products (H_2_, CH_4_, C_2_H_4_, CO, CH_3_CHO and CO_2_) generated from the flash light photo-pyrolysis of the banana peel for 12 s with 5 flash shots at different voltages: 375 V-pulse, 475 V-pulse and 575 V-pulse, which corresponds to a pulse power energy of 6.5, 9.7 and 13.1 J cm^−2^ per flash shot, respectively. According to these data, it can be seen that the gas yield increases with the voltage and power energy since a higher temperature is reached (Fig. S3†), except for CH_3_CHO (Fig. 3e) that presents a higher yield at the 475 V-pulse, and this finding also evidences that liquid compounds that must be generated in the first flash shots are further photo-pyrolyzed to gases at higher power energies in the last flash shots. Here, higher flash light irradiation at the 575 V-pulse provides a simulated temperature on the surface of steel of 500 °C after 12 s, although experimental temperatures are likely to be higher generating mainly H_2_ and CO (Fig. 3a and d).

**Fig. 4:**
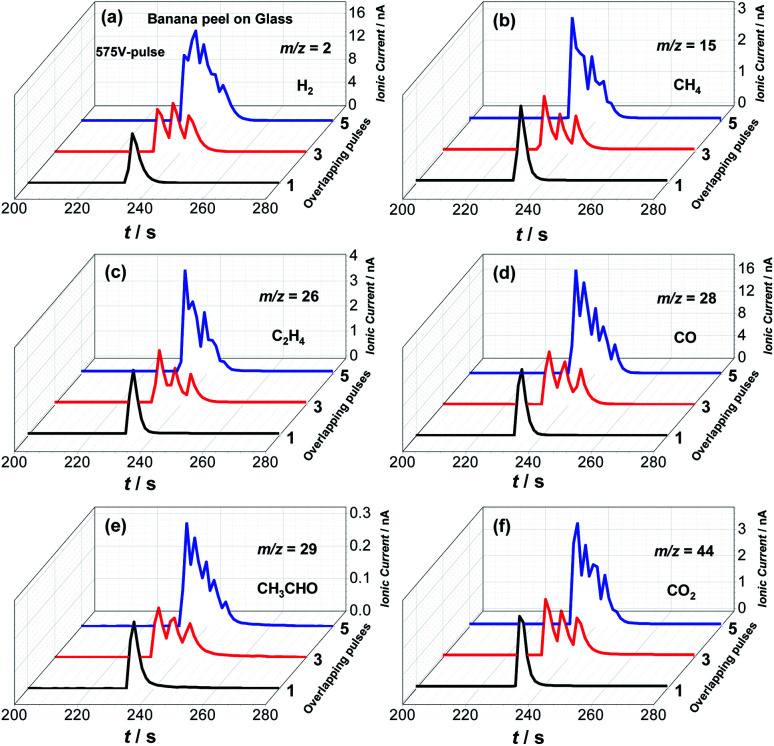
(a–f) Ionic currents for *m*/*z* 2 (H_2_), 15 (CH_4_), 26 (C_2_H_4_), 28 (CO), 29 (CH_3_CHO) and 44 (CO_2_) obtained during the photo-pyrolysis of banana peel by flash light irradiation with a 575 V-pulse at different flash shots: 1, 3 and 5.

Additional tests were carried out to investigate the effects of different flash pulses and shots parameters as illustrated in Fig. 4, S4 and S5,† respectively. Fig. 4 presents MS ionic signals for H_2_, CH_4_, C_2_H_4_, CO, CH_3_CHO and CO_2_ generated from the photo-pyrolysis of the banana peel powder by flash light irradiation with a 575 V-pulse by using 1, 3 and 5 flash shots. Here, it is clear that increasing the number of flash shots also increases the gas production since the sum of consecutive single flash shots reaches higher temperatures for a longer period of time as illustrated in Fig. S4.†

As discussed above, consecutive single flash shots must also promote secondary photo-pyrolysis of liquids and volatile products generated in the first flash shots, and thus photo-thermal biomass splitting from white flash light irradiation can be considered not only as a rapid but also powerful protocol to maximize the gaseous phase from biomass photo-pyrolysis. Additional measurements were also performed to investigate the influence of consecutive flash shot series in the gas yield from the banana peel pyrolysis as presented in Fig. S5,† and here 5 consecutive series of 5 flash shots were carried out from 2 mg of banana peel powder with an interval time between the series of 5 flash shots of 160 s. According to these data, it is clear that most of the syngas content is generated in the first flash light shot series, and hence our choice to carry out measurements in 12 seconds.

**Fig. 5:**
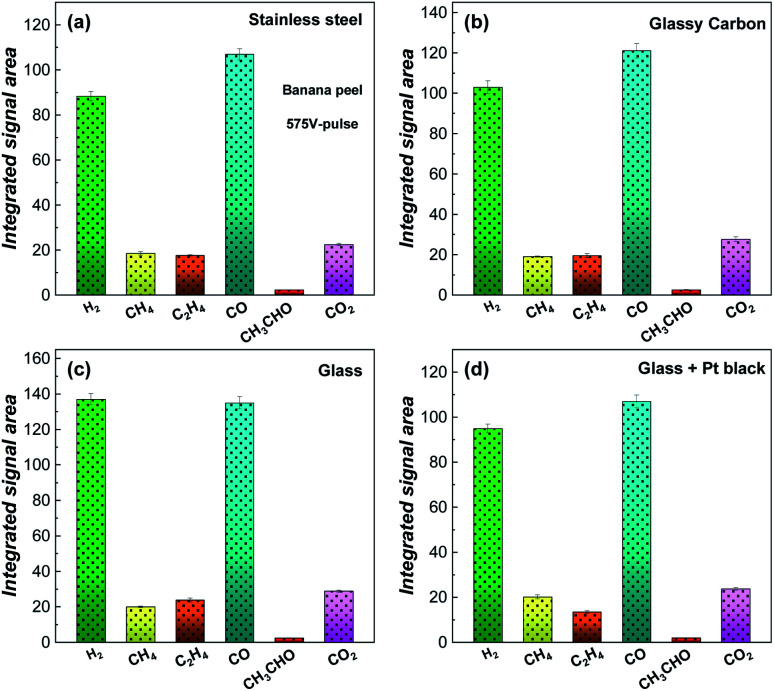
Integrated ionic currents for *m*/*z* 2 (H_2_), 15 (CH_4_), 26 (C_2_H_4_), 28 (CO), 29 (CH_3_CHO) and 44 (CO_2_) generated from the banana peel photo-pyrolysis by flash light irradiation with a 575 V-pulse and 5 flash shots and different substrates: (a) stainless steel, (b) glassy carbon, (c) glass and (d) glass + Pt black.

Tests were performed from different substrates with different light absorptions, thermal and electronic conductivities (stainless steel, glass, glassy carbon and glass + Pt black micro-particles) to investigate the effects of thermo- and electrocatalytic processes. Fig. 5 presents integrated MS peak areas for H_2_, CH_4_, C_2_H_4_, CO, CH_3_CHO and CO_2_ obtained from the banana peel pyrolyzed with a 575 V-pulse and 5 flash shots. Regardless of the substrate used, hydrogen and carbon monoxide are generated as the main products and the process performed on a glass substrate (Fig. 5c) provided a higher yield of gas products, in particular for H_2_, compared for example with a glassy carbon substrate (Fig. 5b). These results suggest that the flash light irradiation promotes a photo-thermal process directly from the biomass. Substrate thermo- and electro-effects (Fig. 5a and b) and catalytic effects (Fig. 5d) seem not to increase the yield of the biomass photo-pyrolysis into gas products. Hence, the reactions can be considered mainly as a high-temperature photolysis process since molecules like lignin, cellulose, and hemicellulose (from natural biomass) are broken into small molecules (H_2_, CO, CH_4_, CO_2_, and C_2_H_4_) by using the light absorption of the biomass source itself, which must promote high energy reactions not on the solid phase, but directly in the volatile phases generated in the first flash light shots.

Photo-thermal biomass splitting from white flash light irradiation can, of course, also be applied to photo-pyrolyze different natural biomass wastes. Results from corncob, orange peel, coffee bean, coconut shell, *etc.,* in Fig. S6 to S10† also evidence that H_2_, CH_4_, C_2_H_4_, CO, CH_3_CHO, CO_2_ gases and porous biochar are generated as the main products.

**Fig. 6:**
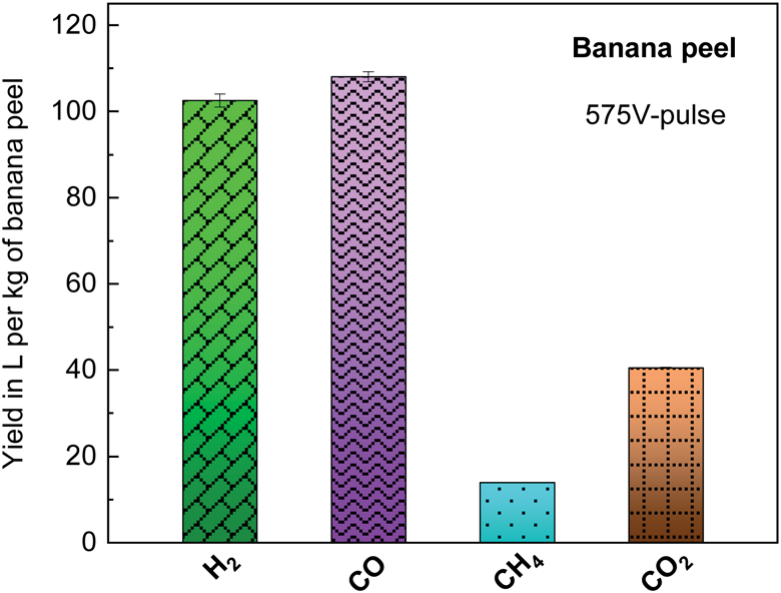
Yield in liters per kg of biomass of the by-products generated from banana peel photo-pyrolysis by flash light irritation on a glass substrate with a 575 V-pulse and 5 flash shots.

Fig. 6 presents the yield in liters per kg of banana peel of the H_2_, CO, CH_4_ and CO_2_ products, estimated by gas chromatography analysis as illustrated in Table S3,† generated during the banana peel photo-pyrolysis on a glass substrate with a 575 V-pulse and 5 flash shots. Here, as already discussed above, gaseous/volatile and solid compounds are generated as the main by-products, and it is important to mention that the high temperature with a high heating rate, shorter residence period of reaction and small biomass granulometry increase the gaseous products and decrease the bio-oil and solid biochar contents. In particular, H_2_, CO and CH_4_ correspond to 15.4 wt% per kg of dried biomass with *ca.* 8 wt% CO_2_.

Secondary photo-pyrolysis from water, gases and bio-oils eventually generated in the first flash shots are likely to be pyrolyzed mainly into gases in the remaining flash shots, each starting with a high power microflash (Fig. 1), as can be seen in the equations given in the ESI,† and therefore all those reactions increase even more the yield of gases, in particular hydrogen and carbon monoxide.

The light energy consumption for the biomass photo-pyrolysis from flash light irradiation is *ca.* 5.2 MJ kg^−1^ of banana peel. As no measurements of the electrical consumption of the photonic curing unit were performed, it is difficult to compare this process with conventional methods that evidence an energy consumption for pyrolysis of 780 MJ per kg of banana peel as described by Tahir *et al.*^21^ In their work, banana peel pyrolysis was performed at different heating rates to 800 °C generating 72% of the total gaseous compounds such as C=C, CH_3_COOH, and CO_2_. Also, 70% of the total energy was related to liquid products, and 24% solid products and 6% gas. The high heating value (HHV) for banana peel, *i.e.* 18.87 MJ kg^−1^, was recovered at a level of 16.41 MJ kg^−1^ (87% efficiency).

In the present work, the hydrogen atom content of the dried banana peel was 4.94 wt%, of which 18.6 wt% was recovered as H_2_ gas, representing 102.5 L that may generate a high heating value of 1.3 MJ kg^−1^. The solid biochar component represents 33.0 wt% and provides a HHV of 6.05 MJ kg^−1^. All in all, the photo-pyrolysis process considering solid biochar, H_2_, CO and CH_4_ produces 9.29 MJ kg^−1^, which corresponds to a positive energy balance (HHV *versus* light energy) of 4.09 MJ kg^−1^, without significant production of CO_2_. The HHV measured for banana peel was 16.45 MJ kg^−1^ and the flash light irradiation process recovered 56% of the total energy as gases and biochar. However, this process is still limited by the flash light irradiation system as it provides an electrical efficiency of *ca.* 28%.

## Conclusions

Natural biomass splitting into valuable gases and biochar by using a flash light irradiation process was introduced as a smart, rapid and eco-friendly approach to transform dried natural biomass wastes into energy with carbon mitigation. Here, the high-power energy from a xenon flash light promotes photo-thermal chemical reactions at high temperatures for a very short residence time (*ca.* 14.5 ms per flash shot). Small biomass particle sizes (20 μm), quantities (2–10 mg) and a thin biomass film (50 μm) were also adopted to maximize the light absorption and then the heating generation maximizing the gas phase yield for H_2_, CO and CH_4_ (15.4 wt% of valuable gas), solid phase biochar (33 wt%) and only 8 wt% CO_2_. This approach does not require any catalyst to promote the photo-thermal biomass conversion being performed in a normal stainless-steel reactor without pressure and by using a standard borosilicate glass window and under an inert atmosphere, the biomass conversion yield is mainly limited by the exposed area, here in the range of cm^2^; however it can be extended for industrial applications. This process has also the advantage to minimize the light energy consumption since only 5.2 MJ is required to pyrolyze 1 kg of biomass promoting the production of valuable gases, in particular hydrogen with *ca.* 100 L and providing an energy balance output of 4.09 MJ kg^−1^ of dried biomass. It also produces conductive porous carbon for further industrial applications or soil amendment. Additionally, carbon can be stored and contribute to a carbon capture strategy.

It is worth mentioning that the present approach is based on surface reactions and therefore does not require additional energy to heat the whole reactor. Admittedly, a complete life cycle analysis of the process and/or detailed exergy calculations must be done.

In conclusion, the present work provides the first steps of a new route for hydrogen production from biomass and eventually from other industrial wastes such as tyres, and a rather efficient way to capture carbon. It opens the way to solar photo-pyrolysis.

## Data availability

All the data have been included in the ESI.†

## Author contributions

All authors designed collectively the experiments, discussed the results and contributed to the manuscript. W. O. S. performed the MS, GC, Raman, XPS experiments and their respective data analyses. B. N. performed GC and SEM experiments and related data analysis. All authors designed collectively the experiments, discussed the results and contributed to the manuscript. W. O. S. performed the MS, GC, Raman, XPS experiments and their respective data analyses. B. N. performed GC and SEM experiments and related data analysis. All authors designed collectively the experiments, discussed the results and contributed to the manuscript. W. O. S. performed the MS, GC, Raman, XPS experiments and their respective data analyses. B. N. performed GC and SEM experiments and related data analysis.

## Conflicts of interest

There are no conflicts to declare.

## Supplementary Material

SC-013-D1SC06322G-s001
