# Supplementary material for: Banana split: biomass splitting with flash light irradiation
Source: Chem Sci. 2022 Jan 25;13(6):1774–9. doi: 10.1039/d1sc06322g (PMC8827125; doi:10.1039/d1sc06322g)
Supplement: SC-013-D1SC06322G-s001 [file SC-013-D1SC06322G-s001.pdf]

Electronic Supplementary Material (ESI) for Chemical Science.
This journal is © The Royal Society of Chemistry 2022

Supplementary information

Banana split: Biomass splitting with flash light irradiation

Wanderson O. Silva,*[a] Bhawna Nagar,[a] Mathieu Soutrenon,[b] and Hubert H. Girault [a]

[a]*Laboratory of Physical and Analytical Electrochemistry, École Polytechnique Fédérale de Lausanne (EPFL) Valais Wallis, Rue de l'Industrie 17, CH-1950 Sion, Switzerland.*

[b]*Institute of Systems Engineering, HES-SO Valais- Wallis, CH-1950 Sion, Switzerland.*

Corresponding author.

E-mail address: wanderson.oliveiradasilva@epfl.ch

Table S1 – Instrumental conditions of the micro GC analyses used in this study.

Agilent 490 Micro GC Instrument Conditions	Channel 1: MS5A, 10 m, TCD	Channel 2: PPU, 10 m, TCD	Channel 3: MS5A, 10 m, TCD
Column temperature	100 °C	100 °C	100 °C
Carrier gas	Helium, 200 kPa	Helium, 200 kPa	Argon, 200 kPa
Injector temperature	110 °C	110 °C	110 °C
Injection time	150 ms	150 ms	150 ms
Detector sensitivity	Auto	Auto	Auto
Sample line temperature	110 °C	110 °C	110 °C
Sampling mode	Continuous flow	Continuous flow	Continuous flow
Sampling time	30 s	30 s	30 s

MS5A: Molecular Sieve 5A; PPU: PoraPLOT U; TCD: Thermal Conductivity Detector.

Table S2 – Elemental composition of the banana peel.

Element	wt. %	Std. Dev.
C	41.360	0.044
H	4.939	0.022
N	1.300	0.030
S	0.101	0.011
O*	52.300	0.086

*Calculated by difference.

Figure S1 - (a) PulseForge 1300 photonic curing system (Novacentrix, US), b) overview of the sample table/support and Xenon flash head, (c) power supply, (d) banana peel, (e) biochar generated after photo-pyrolysis at 575V-pulse and 5 flash shots and (f) typical emission spectrum of the Xenon lamp.

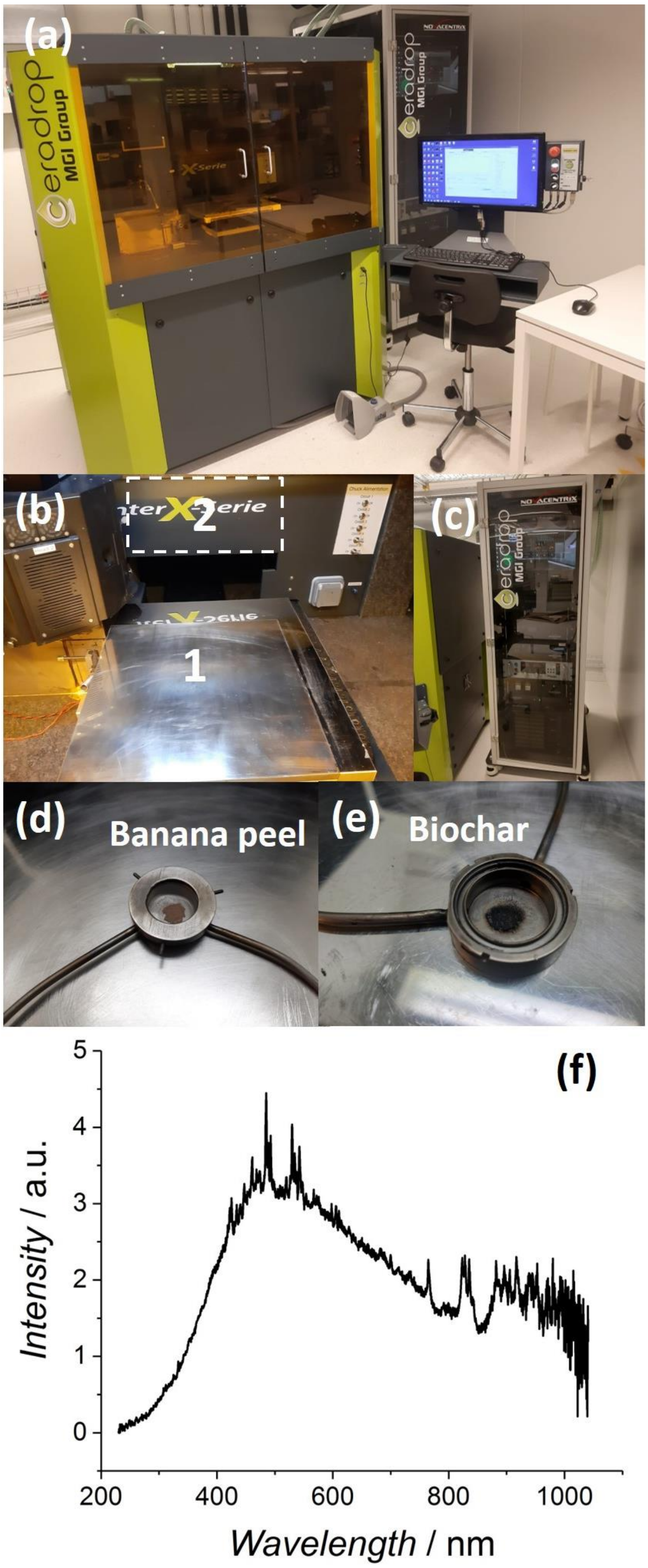

Figure S2 - (a) Raman and XPS spectra of the biochar synthesized from banana peel by photo-pyrolysis at 575V-pulse and 5 flash shots.

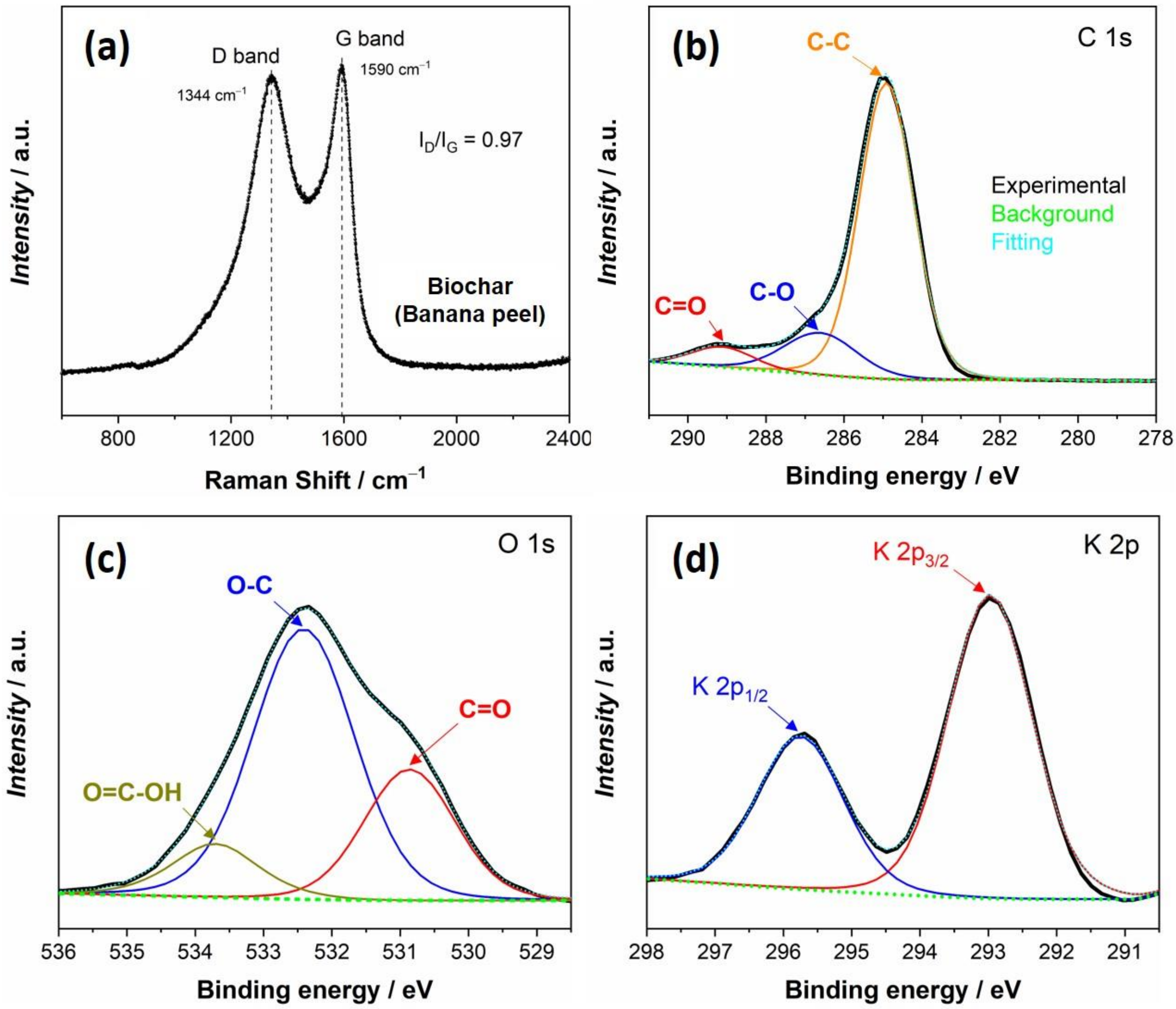

Figure S3 – Simulated temperature profiles obtained during the flash light irradiation of the banana peel powder on glass substrate from 5 flash shots at: (a) 375V-pulse, (b) 475V-pulse and (c) 575V-pulse.

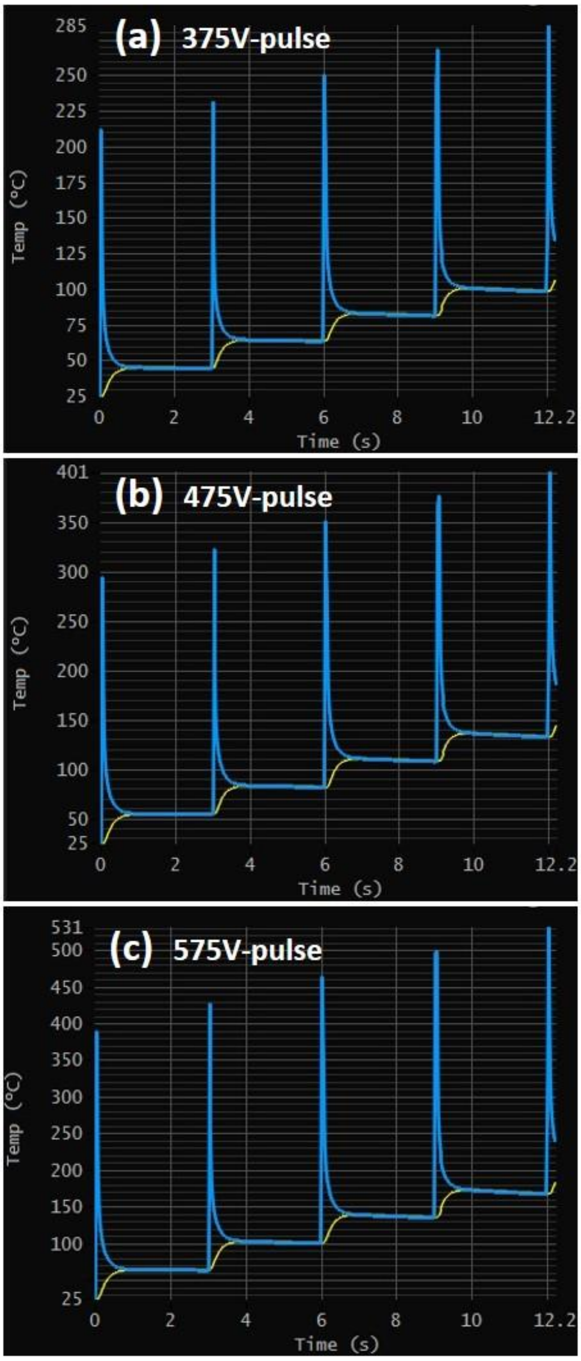

Figure S4 – Simulated temperature profiles obtained during the flash light irradiation of the banana peel powder on glass substrate at 575V-pulse from different flash shots: (a) 1, (b) 3 and (c) 5.

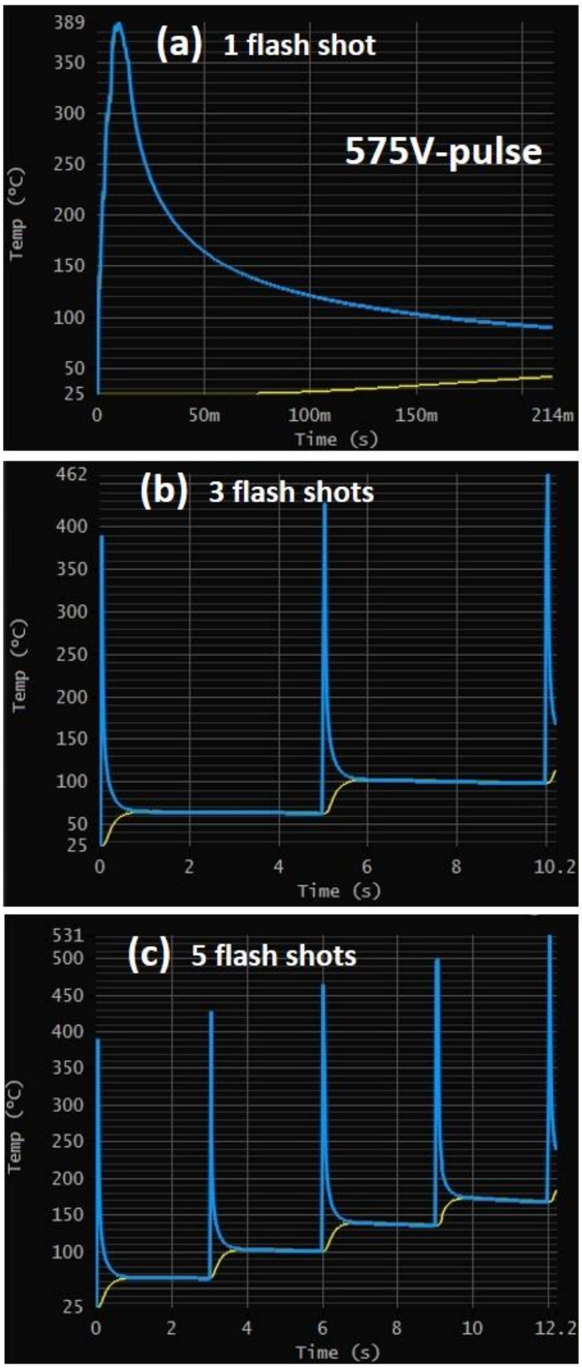

Figure S5 - Ionic currents for m/z 2 (H_2), 15 (CH_4), 26 (C_2H_4), 28 (CO), 29 (CH_3CHO) and 44 (CO_2) obtained during the photo-pyrolysis of banana peel by flash light at 575V-pulse from a total of 25 flash shots or overlapping pulses (OP) divided into 5 consecutive tests of 5 consecutive flash shots.

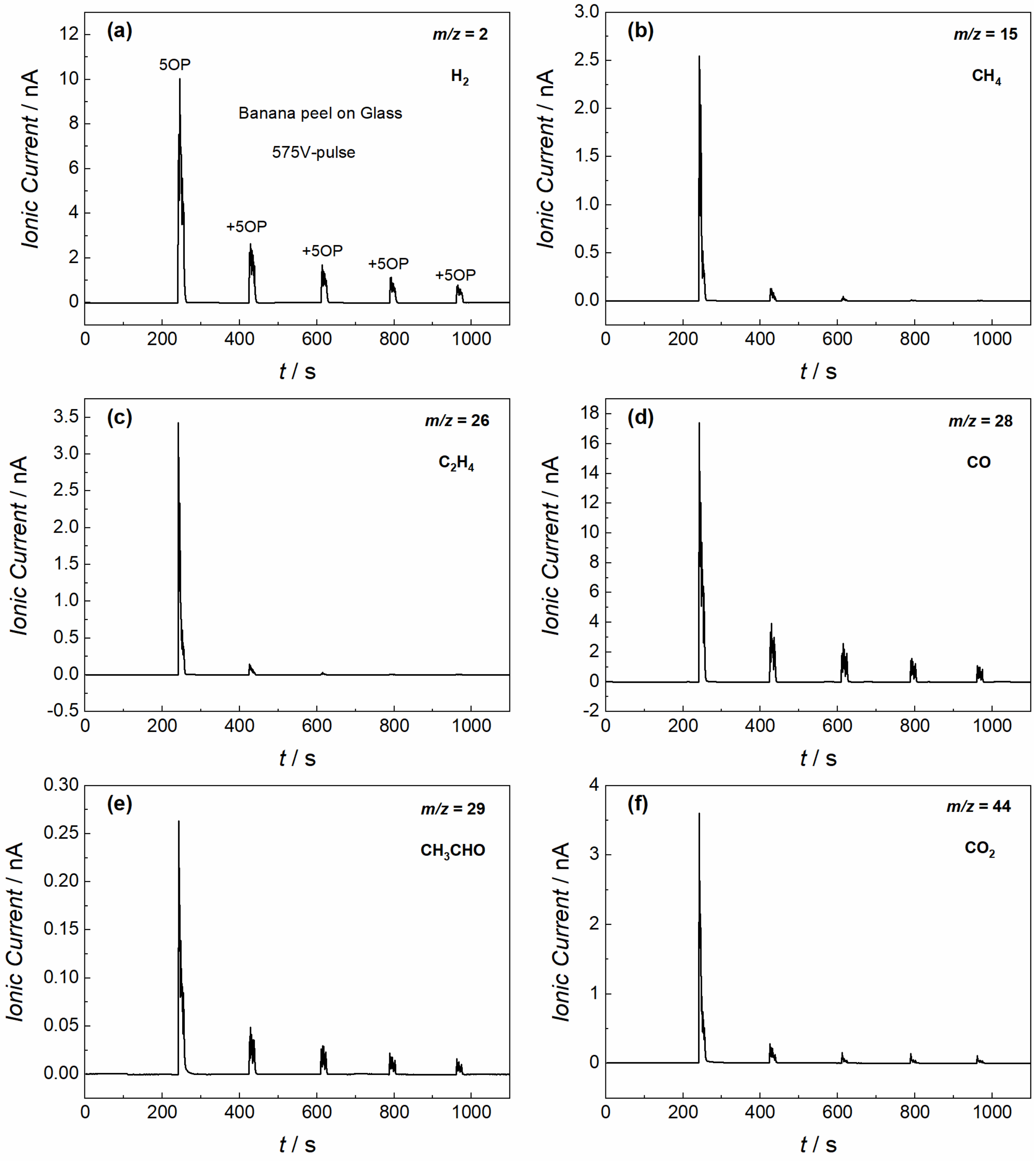

Figure S6 - Ionic currents for m/z 2 (H_2), 15 (CH_4), 26 (C_2H_4), 28 (CO), 29 (CH_3CHO) and 44 (CO_2) obtained during the photo-pyrolysis of corncob, orange peel, coffee bean and coconut shell by flash light irradiation at 575V-pulse and 5 flash shots.

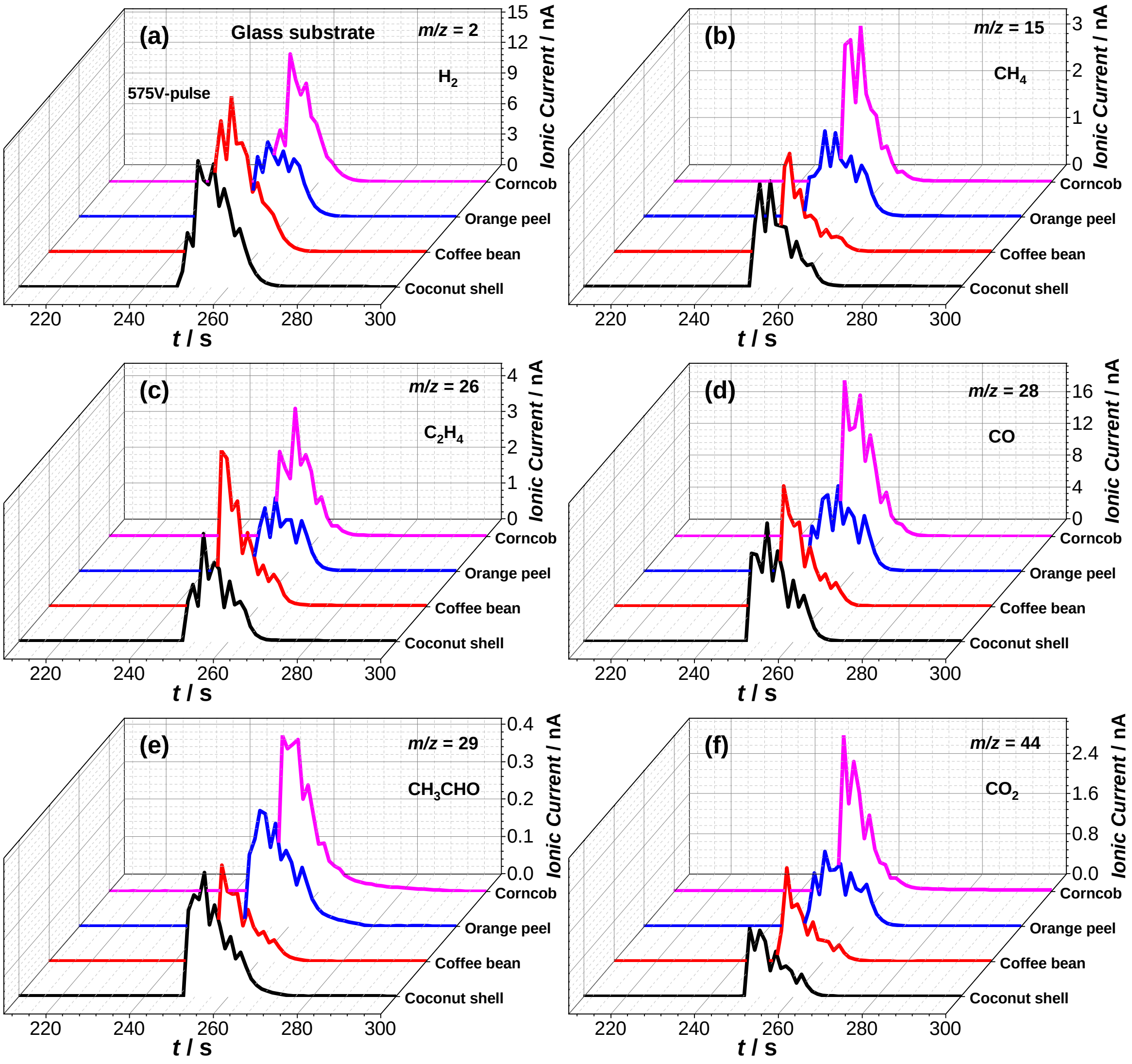

Figure S7 - Scanning electron microscopy images of (a) corncob powder and (b) biochar generated from photo-pyrolysis at 575V-pulse and 5 flash shots and its respective (c) Raman spectrum.

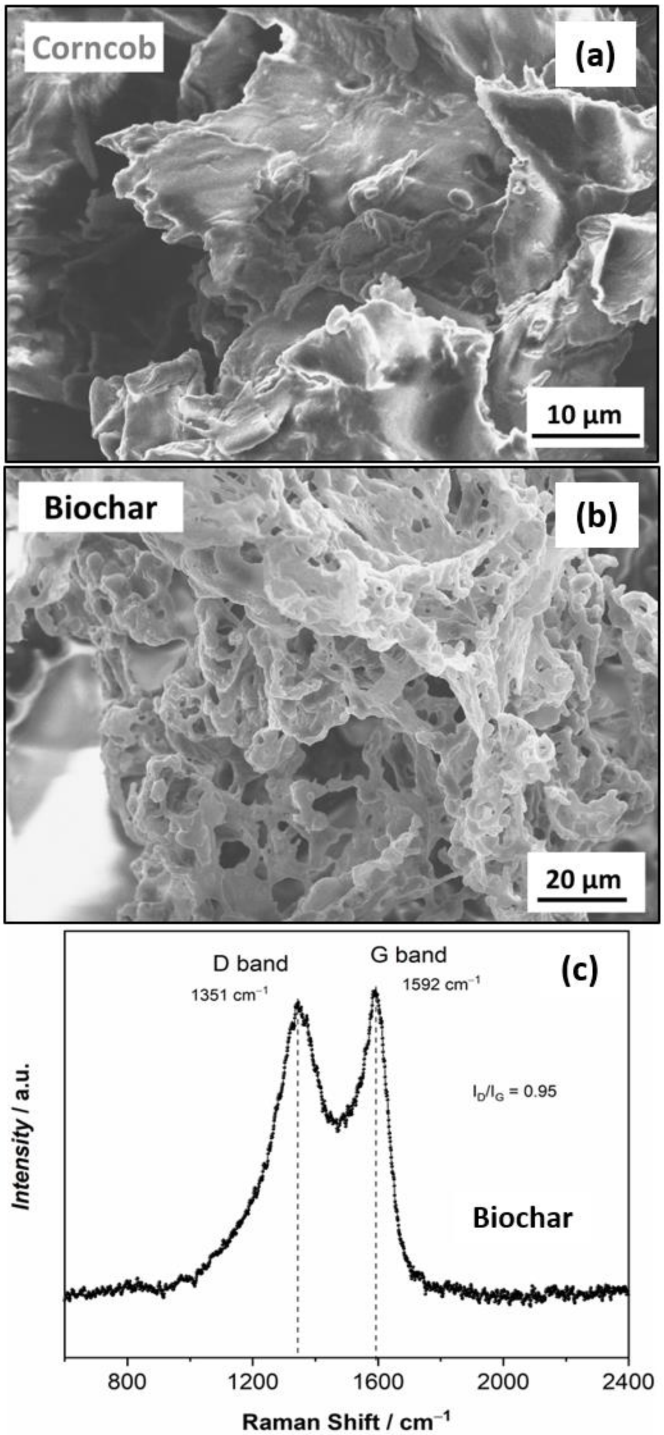

Figure S8 - Scanning electron microscopy images of (a) orange peel powder and (b) biochar generated from photo-pyrolysis at 575V-pulse and 5 flash shots and its respective (c) Raman spectrum.

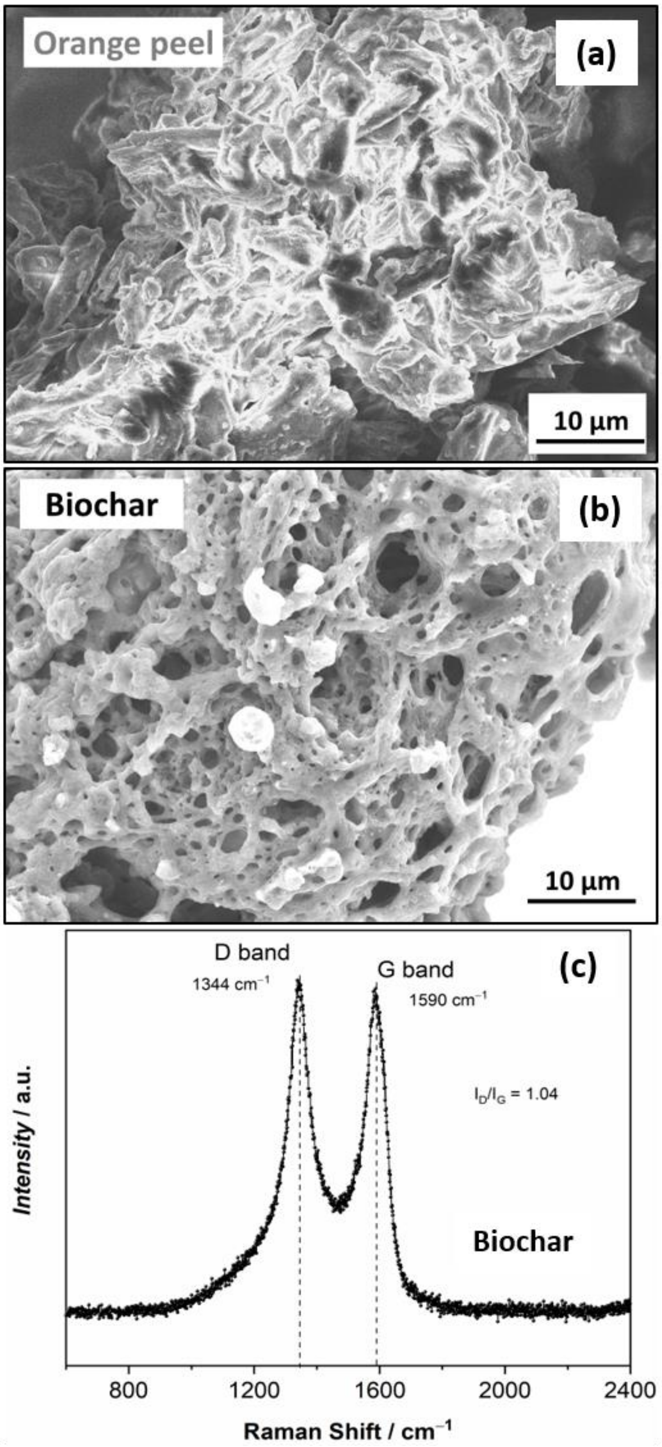

Figure S9- Scanning electron microscopy images of (a) coffee bean powder and (b) biochar generated from photo-pyrolysis at 575V-pulse and 5 flash shots and its respective (c) Raman spectrum.

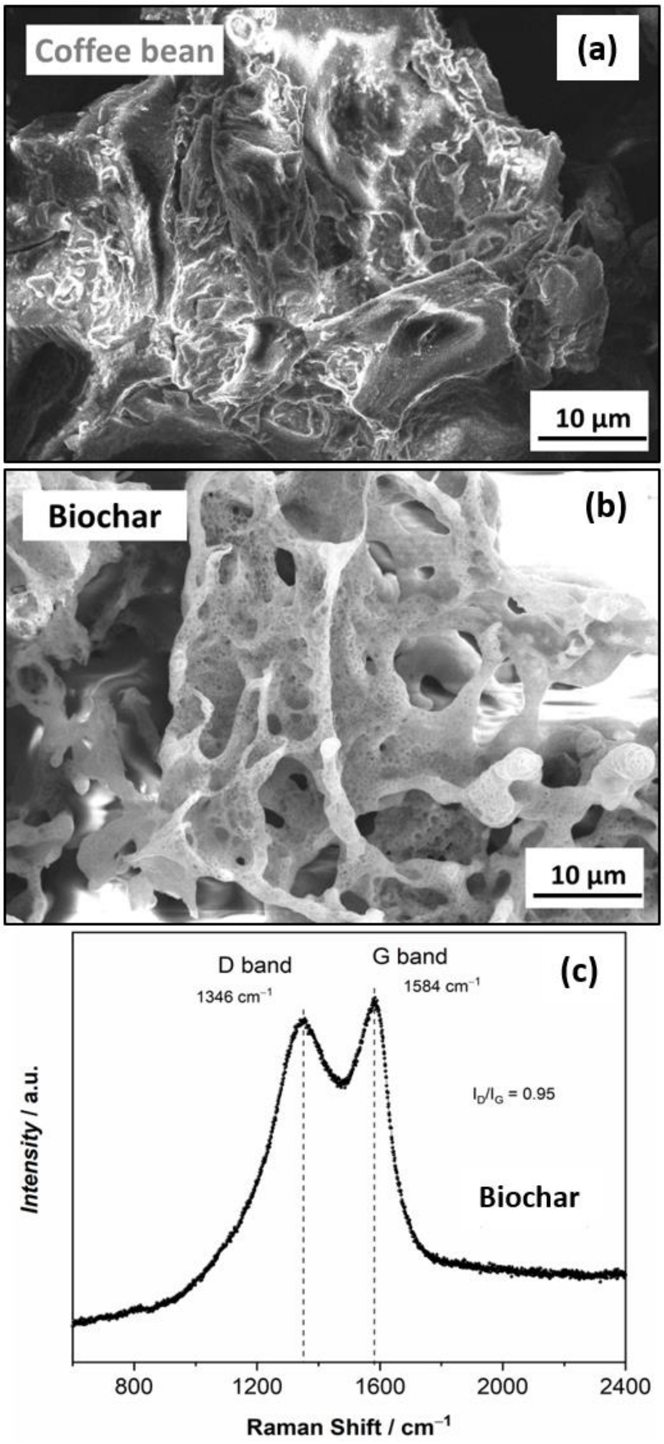

Figure S10 - Scanning electron microscopy images of (a) coconut shell powder and (b) biochar generated from photo-pyrolysis at 575V-pulse and 5 flash shots and its respective (c) Raman spectrum.

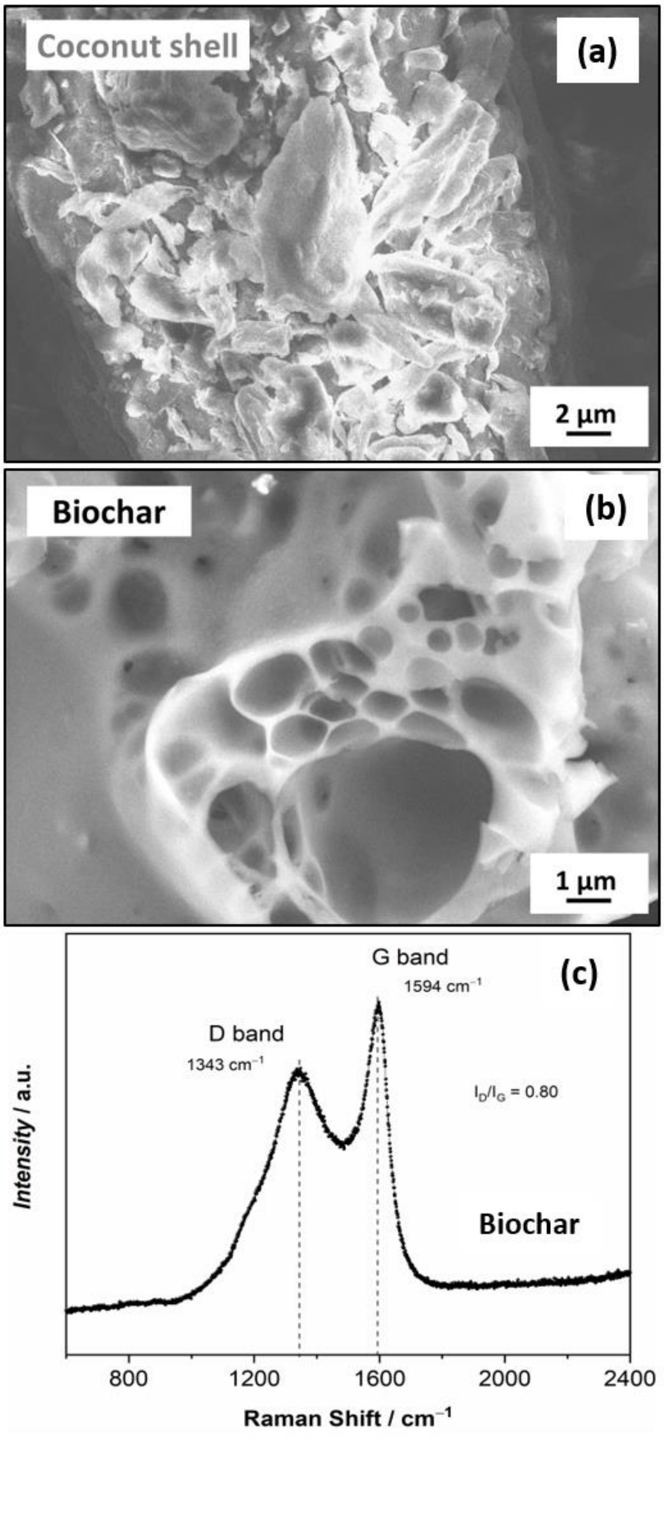

Table S3 – Yield calculations of the gases generated from banana peel photo-pyrolysis by flash light irradiation on glass substrate at 575V-pulse and 5 flash shots.

Gases	v/v (%) – Micro GC	Gas volume from 10 mg of biomass (L)	Density (g L^{-1})[a]	Gas weight from 10 mg of biomass (g)	Gas weight per kg of biomass (g kg^{-1})	Gas volume per kg of biomass (L kg^{-1})
H_2	20.5	0.001025	0.0898	0.0000920	9.2	102.5
CH_4	2.8	0.000140	0.7167	0.0001003	10.0	14.0
CO	21.6	0.001080	1.2501	0.0013501	135.0	108.0
CO_2	8.1	0.000405	1.9768	0.0008006	80.1	40.5

[a]https://onlinelibrary.wiley.com/doi/pdf/10.1002/9781118131473.app2

Secondary pyrolysis from water, gases and tar/bio-oils

Biomass + Flash Light Irradiation (fast heating) → C + H_2 + CO + CH_4 + CO_2 + C_2H_4 + CH_3CHO + H_2O + primary tar (Pyrolysis)

Primary tar + Flash Light Irradiation (fast heating) → H_2 + CO + CH_4 + CO_2 + C_2H_4 + CH_3CHO + secondary tar (primary tar cracking, Pyrolysis)

Secondary tar + Flash Light Irradiation (fast heating) → H_2 + CO + CH_4 + CO_2 + C_2H_4 + CH_3CHO + tertiary tar (secondary tar cracking, Pyrolysis)

Tertiary tar + Flash Light Irradiation (fast heating) → C + H_2 + CO (tertiary tar cracking, Pyrolysis)

$2CH_4 + O_2 \rightarrow 2CO + 2H_2$ (Combustion)

$CH_4 + CO_2 \rightarrow 2CO + 2H_2$ (Dry reforming)

$C_2H_4 + 2CO_2 \rightarrow 4CO + 2H_2$ (Dry reforming)

$CH_4 + H_2O \rightarrow CO + 3H_2$ (Steam reforming)

$C_2H_4 + 2H_2O \rightarrow 2CO + 4H_2$ (Steam reforming)

$CO + H_2O \rightarrow CO_2 + H_2$ (Water gas shift)

$C + O_2 \rightarrow CO_2$ (Carbon oxidation)

$2C + O_2 \rightarrow 2CO$ (Partial oxidation)

$C + CO_2 \rightarrow 2CO$ (Boudouard reaction)

$C + H_2O \rightarrow CO + H_2$ (Water gas reaction)
